# Protective effect of zinc preconditioning against renal ischemia reperfusion injury is dose dependent

**DOI:** 10.1371/journal.pone.0180028

**Published:** 2017-07-07

**Authors:** Kenny Rao, Kapil Sethi, Joseph Ischia, Luke Gibson, Laurence Galea, Lin Xiao, Mildred Yim, Mike Chang, Nathan Papa, Damien Bolton, Arthur Shulkes, Graham S. Baldwin, Oneel Patel

**Affiliations:** 1 Department of Surgery, The University of Melbourne Victoria, Australia; 2 Department of Urology Austin Health, Victoria, Australia; 3 Department of Anatomical Pathology, Austin Health, Victoria, Australia; University Jean MONNET of SAINT-ETIENNE, UNITED STATES

## Abstract

**Objectives:**

Ischemia-reperfusion injury (IRI) is a major cause of acute kidney injury and chronic kidney disease. Two promising preconditioning methods for the kidney, intermittent arterial clamping (IC) and treatment with the hypoxia mimetic cobalt chloride, have never been directly compared. Furthermore, the protective efficacy of the chemically related transition metal Zn^2+^ against renal IRI is unclear. Although Co^2+^ ions have been shown to protect the kidney via hypoxia inducible factor (HIF), the effect of Zn^2+^ ions on the induction of HIF1α, HIF2α and HIF3α has not been investigated previously.

**Materials and methods:**

The efficacy of different preconditioning techniques was assessed using a Sprague-Dawley rat model of renal IRI. Induction of HIF proteins following Zn^2+^ treatment of the human kidney cell lines HK-2 (immortalized normal tubular cells) and ACHN (renal cancer) was measured using Western Blot.

**Results:**

Following 40 minutes of renal ischemia in rats, cobalt preconditioning offered greater protection against renal IRI than IC as evidenced by lower peak serum creatinine and urea concentrations. ZnCl_2_ (10 mg/kg) significantly lowered the creatinine and urea concentrations compared to saline-treated control rats following a clinically relevant 60 minutes of ischemia. Zn^2+^ induced expression of HIF1α and HIF2α but not HIF3α in HK-2 and ACHN cells.

**Conclusion:**

ZnCl_2_ preconditioning protects against renal IRI in a dose-dependent manner. Further studies are warranted to determine the possible mechanisms involved, and to assess the benefit of ZnCl_2_ preconditioning for clinical applications.

## Introduction

Partial nephrectomy (PN) using minimally invasive (laparoscopic or robotic-assisted) surgery is now the gold standard treatment for small kidney cancers. PN has been shown to provide equivalent cancer control and decreased risk of chronic kidney disease [1]. However, clamping the renal artery during PN leads to renal ischemia reperfusion injury (IRI) and renal dysfunction. IRI describes a complex array of tissue-destructive processes that is one of the key causes of acute kidney injury (AKI) following PN. IRI is also a serious complication of human organ transplantation.

The consequences of AKI in surgical patients are potentially disastrous with a short term 5–8 fold increase in post-operative 30 day mortality and a long term risk of CKD in 5–17% of patients [2]. Therefore, the ischemia time must be kept as short as possible. Current clinical data support a “safe” warm ischemia time of 25 minutes and a cold ischemia time (when the kidney is placed on ice slush) of 35 minutes (and up to 2 hours can be tolerated) [2]. However, cold ischemia is impractical in laparoscopic surgery. The overwhelming concern amongst urologists about the deleterious effect of IRI means that less than 25% of resections are done by PN and over 75% by radical nephrectomy [3]. An intervention which could extend the critical ischemia time and minimize the possibility of AKI would dramatically increase the proportion of cancers that could be treated with nephron-sparing PN rather than radical nephrectomy [1].

Ischemic preconditioning in general involves exposing an organ to sub-lethal ischemia to protect it against subsequent ischemic injury [4]. Intermittent clamping (IC) is one method of ischemic preconditioning in which the organ to be protected is subjected to multiple short cycles of ischemia and then reperfusion, whereby it is postulated that the tissue will upregulate protective mechanisms before the main ischemic insult. Although IC has shown promise in preclinical models of liver and cardiac ischemia, it has not been studied in the human kidney. Newer chemical preconditioning methods, such as treatment with cobalt chloride (CoCl_2_,) have been shown to protect the kidney against IRI via hypoxia inducible factor (HIF) [5]. Although subcutaneous injection of cobalt has been shown to ameliorate renal IRI in a rat model, cobalt is unsuitable for human use because of its toxic effects [6]. Salts of zinc, a chemically related transition metal, are commonly ingested by humans and have also been shown to either exacerbate or protect against myocardial or neuronal ischemic injury (reviewed in [7, 8]).

A direct comparison of the efficacy of IC and cobalt preconditioning, either separately or in combination, has not been reported previously. Therefore the aim of this study was to compare the protective ability of IC with CoCl_2_ preconditioning in a rat model of IRI. Having established the proof-of-principle benefit of the hypoxia-mimetic CoCl_2_, the protective ability of zinc chloride (ZnCl_2_) was compared to CoCl_2_ using the same rat model of IRI. Co^2+^ ions inhibit iron-dependent prolyl hydroxylases (PHD) by displacing Fe^2+^ and thus cause accumulation of the active HIF protein. We hypothesized that Zn^2+^ will displace Fe^2+^ in a manner similar to Co^2+^, thereby inhibiting PHD activity [9] and inducing HIFs. Therefore in this study the ability of Zn^2+^ ions to induce HIFs was examined using the immortalized human proximal renal tubule cell line HK-2 and the renal cancer cell line ACHN.

## Materials and methods

### Induction of renal ischemic reperfusion injury

Eight to twelve-week-old adult male Sprague-Dawley rats weighing 250–350g were housed 2 per cage in the Bioresources Research Facility at Austin Health, Melbourne, Australia, and provided standard rat pellets and sterile water *ad libitum*. All procedures were approved by the Animal Ethics Committee of Austin Health (project number 2009/03382 and A2012/04638). The committee is guided by the National Health and Medical Research Council (NHMRC) Australian Code of Practice for the Care and Use of Animals for Scientific Purposes 8th edition (2013). Rats were anaesthetised with 5% Isoflurane in a sealed chamber at an oxygen flow rate of 0.5 L/min. Continuous anaesthesia was maintained on a 37°C heat mat via a specially fitted nose cone at a maintenance concentration of between 0.75–1.5%. Prior to surgery, a baseline 400 μL blood sample was drawn from the tail vein. To establish a single kidney model for preconditioning and ischemia, all rats initially underwent a right nephrectomy via a midline incision. Seven days post right nephrectomy, a second blood sample was collected to ensure a return to baseline levels.

Seven days after initial right nephrectomy and after the standard preparation above, the midline laparotomy wound was re-opened and the left renal pedicle was clamped for either 40 or 60 min using a releasable 3/0 silk tie and bulldog vascular clamps. Ischemia of the kidney was confirmed by a rapid colour change to dark purple. After 40 or 60 min the artery was unclamped and the kidney observed to ensure reperfusion.

### Cobalt chloride and Zinc chloride preconditioning

The rats in the intervention groups were preconditioned with subcutaneous injections of CoCl_2_ or ZnCl_2_ at the indicated dose and time in a maximum volume of 0.5 ml saline prior to renal pedicle occlusion. Control groups received placebo preconditioning with subcutaneous injection of 0.5 mL normal saline.

### IC of the renal artery

**Fig 1:**
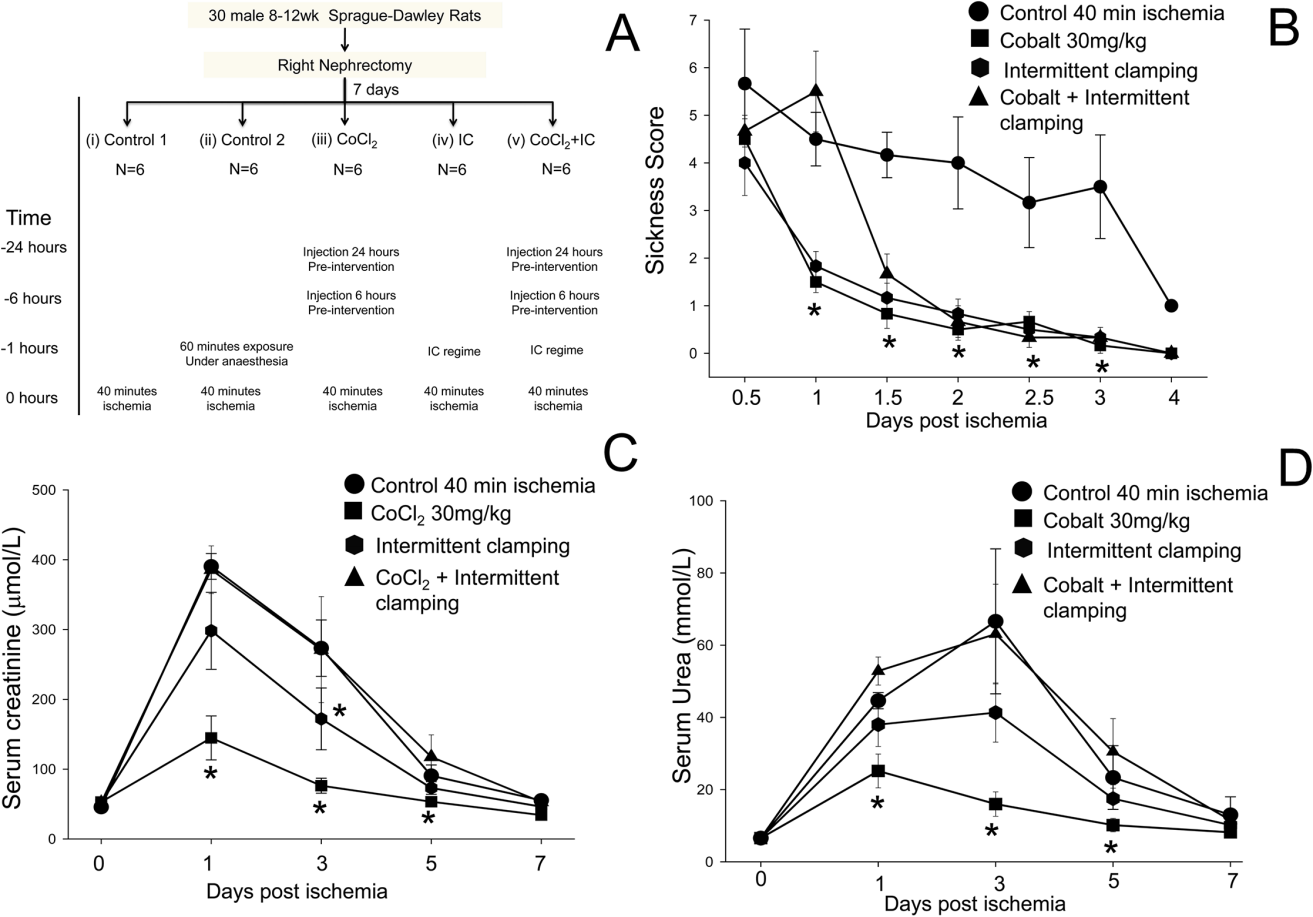
Preconditioning with CoCl_2_ is more effective than intermittent clamping (IC). (A) Experimental design for the *in vivo* study comparing the ability of different preconditioning techniques to protect against IRI in rats. (B) Animal sickness scores in rats treated with saline (control) (●), 30 mg/kg CoCl_2_ (■), IC (⬢) or IC + CoCl_2_ (▲). (C) Serum creatinine concentrations (mean ± SEM (µmol/L)). The rise in serum creatinine was less in rats preconditioned with 30 mg/kg CoCl_2_ than in the saline control group. (D) Serum urea concentrations (mean ± SEM (mmol/L)). *p<0.05 Vs saline treated control.

After standard surgical exposure, the left renal artery was clamped for 5 minutes of ischemia, followed by 10 minutes of reperfusion, over 4 continuous cycles (total time 60 minutes). After completion of this regime, the renal artery was clamped for a further 40 minutes of critical ischemia (Fig 1A).

### Cobalt chloride plus IC of the renal artery

Six rats were injected with CoCl_2_ on the underside flank preoperatively. The left renal artery was then clamped for 5 minutes of ischemia, followed by 10 minutes of reperfusion, over 4 continuous cycles (total time 60 minutes).

### Serum sampling and analysis

Blood samples (400 μL) were collected from the tail vein of the conscious rats into lithium heparin tubes on days 1, 3, 5 and 7. Left kidney was removed for histological analysis, and rats were euthanized by exsanguination on day 7. Blood samples were centrifuged and aliquots stored at -20°C till analysis for creatinine and urea by the Clinical Trials Department, Austin Pathology, Heidelberg, Australia. Creatinine (Jaffé method) and urea (NADH method) in the serum samples were measured on a fully automated Roche Cobas 8000 c702 analyser.

### Sickness score

Animals were monitored daily post-surgery. Scores of 0 (normal), 1 (minor change) or 2 (gross change) were assigned to each of the indicators of pain, distress or suffering and general well-being, which included rough coat, laboured breathing, change in posture, movement, aggression, restlessness or overactivity, or change in body weight as a % of starting weight, and the individual scores were summed for each animal. Health scoring of animals was carried out every 12 hours.

### Histology of kidney sections

Harvested kidneys were fixed overnight in 10% formaldehyde and underwent standard haematoxylin and eosin fixation. Each kidney was analysed semi-quantitatively by a blinded anatomical pathology registrar using two previously published methods of scoring histological damage: (1) the Jablonski acute score [10] (2) the sum of the areas of tubular damage and coagulative necrosis [11].

The morphological changes associated with the Jablonski acute score were graded as follows: Score 0 = normal, Score 1 = Mitoses and necrosis of individual cells; Score 2 = Necrosis of all cells in adjacent proximal convoluted tubules, with survival of surrounding tubules; Score 3 = Necrosis confined to the distal third of the proximal convoluted tubule with a band of necrosis extending across the inner cortex; Score 4 = Necrosis affecting all three segments of the proximal convoluted tubule. Where there were varying Jablonski acute grades across the sections, the case was scored according to the worst grade.

Features associated with acute tubular damage in the proximal and distal convoluted tubules included focal tubular dilation with simplification, flattening and attenuation of the epithelium, loss of the luminal brush border, apoptotic tubular cells and luminal necrotic debris. Areas of coagulative necrosis included areas of loss of renal parenchyma with ghost outlines of cells and structures with sharp demarcation from the adjacent viable tissue. The tubular damage and the coagulative necrosis were assessed individually as a percentage of the area of the whole section and then added according to the acute tubular necrosis (ATN) scoring system [11].

### Mammalian cell lines

The human renal cancer cell line ACHN was cultured in MEM medium plus non-essential amino acids, pyruvate and 7.5% FBS, and the immortalized human proximal renal tubular cell line HK-2 was cultured in DMEM/F12 medium supplemented with bovine pituitary extract and 7.5% FBS, in a humidified incubator at 37°C with 95% air and 5% CO_2_.

### Western blot

Treated cells were lysed with 0.1–0.2 ml pre-boiled sodium dodecyl sulphate (SDS) lysis buffer. Proteins were separated by SDS-polyacrylamide gel electrophoresis, and transferred onto nitrocellulose membrane. Following the transfer membranes were probed with antibodies against HIF1α (monoclonal anti-human, R&D systems) or HIF2α (rabbit anti-human, Cell Signaling) or HIF3α (rabbit anti-human, Abcam) as previously described [12]. Densitometric analysis of the protein bands was performed with MultiGauge software (FujiFilm).

### Statistical analysis

Results are expressed as means ± SEM unless otherwise stated. Statistics were analysed with SigmaStat version 3 (SPSS Inc, Chicago, IL, USA), using t-tests for equally distributed data. Where the data were not normally distributed, were compared with the Mann-Whitney Rank Sum Test. Repeated-measures ANOVA was undertaken to evaluate differences between the two study groups.

## Results

### Cobalt provides superior protection compared to IC post 40 minute ischemia

Rats pre-treated with 30 mg/kg CoCl_2_ according to the protocol shown in Fig 1A showed a significant reduction in sickness score (Fig 1B), in serum creatinine (Fig 1C), and in serum urea (Fig 1D), compared to 40-minute control ischemic rats (p<0.05). The rats had no adverse side effects as a result of injection with CoCl_2_. Although the rats pre-treated with IC showed a significant reduction in sickness score (Fig 1B), the serum creatinine (Fig 1C) values only differed statistically at day 3 and 5, and the serum urea values (Fig 1D) were not statistically different compared to control ischemic animals. The general anaesthetic time was prolonged to 100 minutes in this control group to account for the 60 minutes of anaesthesia delivered during IC, prior to 40 minutes of critical ischemia. There was no statistical difference in serum urea or creatinine between the 40 minute and 100 minute anaesthesia control groups (data not shown). In the six rats that underwent CoCl_2_ preconditioning and intermittent clamping, improvement in the sickness scores was delayed and the serum creatinine and urea were not statistically different from control ischemic animals (Fig 1C and 1D).

### Mortality post 60 minutes of ischemia

**Fig 2:**
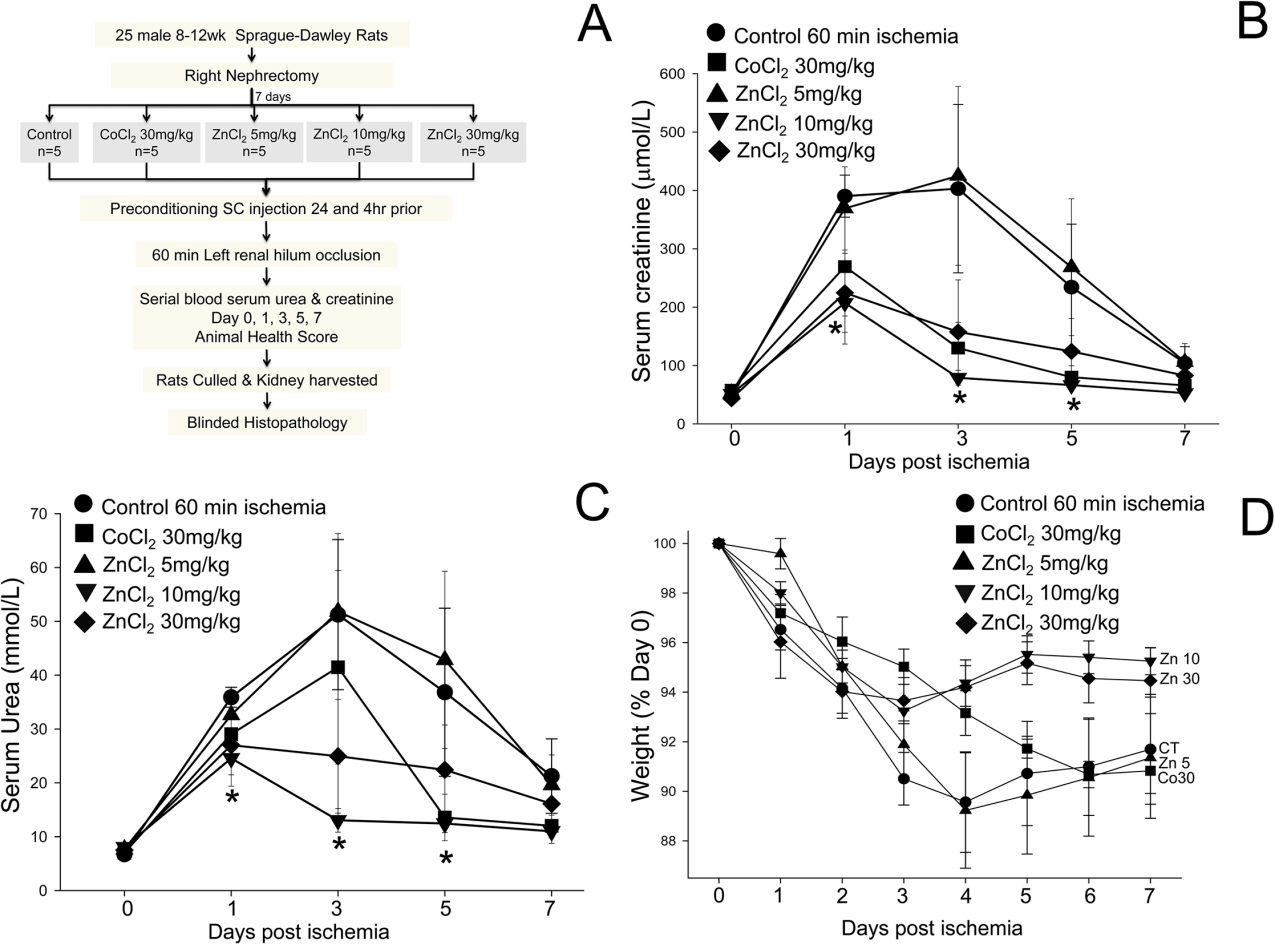
Zinc preconditioning protects against renal ischemia reperfusion injury in rats. (A) Diagram showing the experimental protocol. (B) Serum creatinine concentrations (mean ± SEM (μmol/L)) in rats treated with saline (control) (●), 30 mg/kg cobalt (■), 5 mg/kg ZnCl_2_ (▲), 10 mg/kg ZnCl_2_ (▼) or 30 mg/kg ZnCl_2_ (◆). The rise in serum creatinine was less in rats preconditioned with 10mg/kg ZnCl_2_ than in the saline control group. (C) Serum urea concentrations (mean ± SEM (mmol/L)). The rise in serum urea was less in rats preconditioned with 10 mg/kg ZnCl_2_ than in the saline control group. (D) ZnCl_2_ preconditioning improves the health of rats after IRI. The slope (-0.51) of the line of regression for weight loss in the rats treated with 10 mg/kg ZnCl_2_ was less than the slope for rats treated with 30 mg/kg cobalt (-1.32) or saline (-1.15). *p<0.05 Vs saline treated control. On repeated measures ANOVA, including days one to seven, there was a statistically significant difference between- 10 mg/kg ZnCl_2_ Vs Saline control (p = 0.038).

Previously, a 20% mortality rate following 60 minutes of critical ischemia in rats has been reported [13]. Our data is in agreement with that study as there were 5 mortalities out of the total 25 rats used in our study according to the protocol shown in Fig 2A. As the mortalities were not confined to one particular group but rather extended across all groups (1 in control, 2 in CoCl_2_, 1 in 10 mg/kg ZnCl_2_ and 1 in 30 mg/kg ZnCl_2_ group), 60 minutes of ischemia apparently causes irreversible damage to the kidney in some rats, and ischemia times exceeding 60 minutes would result in an unacceptably high mortality. Therefore, 60 minutes is the ideal ischemic time to test the efficacy of reno-protective agents.

### Zinc preconditioning reduces the rise in serum creatinine and urea concentrations

To explore the beneficial effects of preconditioning with ZnCl_2_, the warm ischemia time was increased to 60 minutes (Fig 2A) and the effects of CoCl_2_ and ZnCl_2_ were compared. Rats treated with 10 mg/kg ZnCl_2_ had a significantly lower rise in serum creatinine and urea after 60 mins ischemia compared to control (p<0.05) (Fig 2B and 2C). On repeated measures ANOVA, including days one to seven, there was a statistically significant difference between 10 mg/kg ZnCl_2_ compared to saline control (p = 0.038). While the serum creatinine and urea were lower in the 30 mg/kg CoCl_2_ and 30 mg/kg ZnCl_2_ groups, the differences did not reach statistical significance. The serum creatinine and urea values in the 5 mg/kg ZnCl_2_ group were indistinguishable from the control group.

### Zinc preconditioning improves the health of animals

Maintenance of body weight is a commonly used marker of animal health during an experimental protocol. Weight loss was observed in all groups post left renal pedicle occlusion (Fig 2D). Following 60 minutes of renal ischemia, the mean percentage weights on day 5, expressed as a percentage of the weight on day 0, were 95 ± 1% and 95 ± 1% in the groups treated with ZnCl_2_ at 10 mg/kg or 30 mg/kg, respectively, compared to 91 ± 2%, 92 ± 0.4% and 90 ± 2% in the control group, and the groups treated with CoCl_2_ at 30 mg/kg or ZnCl_2_ at 5 mg/kg, respectively. Furthermore, a reversal from the peak percentage weight loss occurred on day 4 in the groups treated with ZnCl_2_ at 10mg/kg and 30mg/kg (Fig 1D). Although there was no significant difference between the animal health scores across the groups, zinc preconditioning apparently provides an additional improvement in health outcomes compared to cobalt- or saline-treated rats.

### Zinc administration reduced the tubular damage and necrosis induced by ischemia

**Fig 3:**
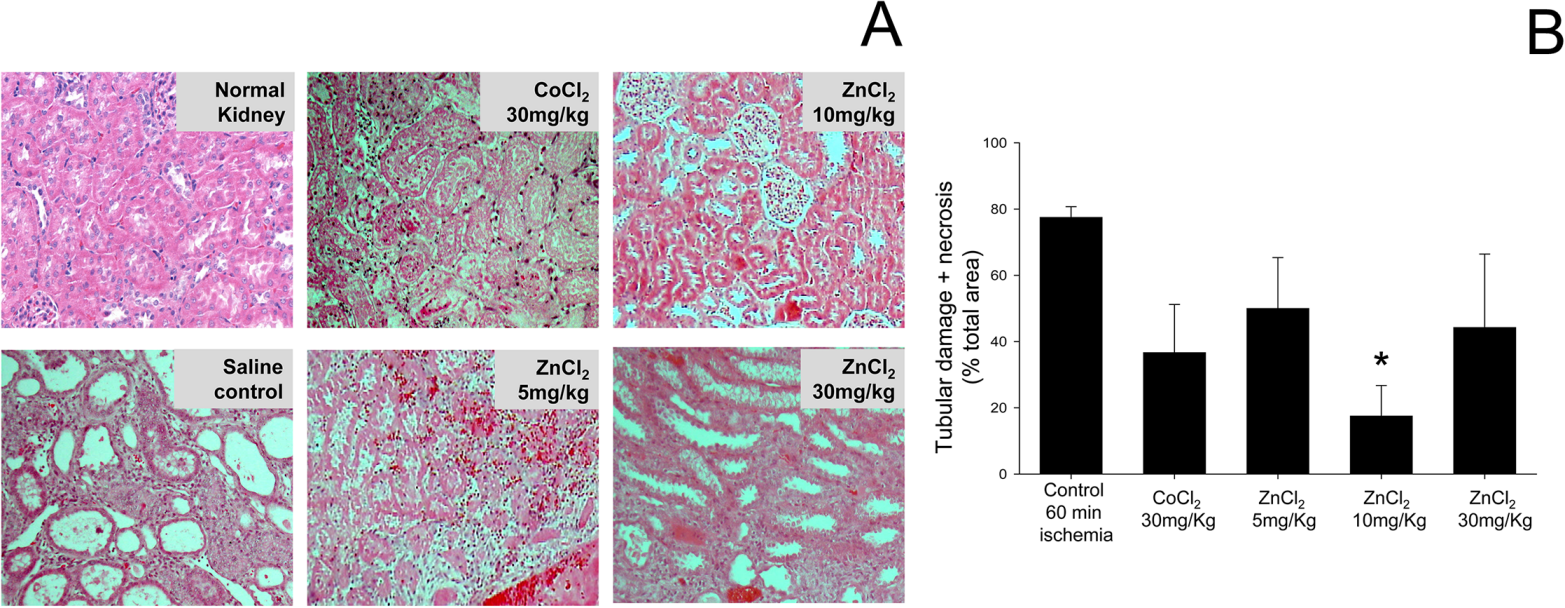
Zinc preconditioning preserves kidney architecture in rats post IRI. (A) Histopathological examination of the kidney by H&E staining 7 days after 60 minutes ischemia. An H&E-stained section of the kidney from a rat treated with 10 mg/kg ZnCl_2_ displays a near-normal cortex with normal glomeruli and no necrosis of individual tubular cells, and has a Jablonski acute score 1. An H&E-stained section of the kidney from a rat treated with 30 mg/kg CoCl_2_ displays complete coagulative tubular necrosis with Jablonski acute score 3. An H&E-stained section of the kidney from a rat treated with saline displays acute tubular injury affecting the distal one third to one half of the proximal convoluted tubules and has a Jablonski acute score 4. (B) Tubular damage and necrosis was significantly (*P < 0.05) reduced in kidneys of rats treated with 10 mg/kg ZnCl_2_. Data are expressed as mean ± SEM for each treatment group.

A grading scale of 0 to 4, as outlined by Jablonski et al. [10], was used to assess the degree of renal tubular necrosis post renal IR injury. Histology of the kidneys in the animals treated with 10mg/kg ZnCl_2_ had the lowest average Jablonski score of all groups representing the most normal looking renal architecture with the least amount of damage (Table 1). Most kidneys showed small areas of coagulative necrosis apart from tubular damage and some showed extensive areas of necrosis, however these necrotic areas may be secondary to global warm ischemia or thrombosis of a segmental artery. A further advantage of the acute tubular injury and necrosis score[11] is that it gives a semi-quantitative numerical score as opposed to just a grade. The acute tubular damage ranged from 2% to 80% and the coagulative necrosis ranged from 0% to 65% across the groups. As shown in Fig 2B, the control group with 77.5 ± 3% had the most acute tubular damage and coagulative necrosis as compared to the other groups. Preconditioning with 10mg/kg ZnCl_2_ significantly (p<0.05) reduced the tubular damage and coagulative necrosis to 18 ± 9%. Although there was a trend for a reduction in tubular damage and coagulative necrosis in the 5mg/kg ZnCl_2_, 30mg/kg ZnCl_2_ and 30mg/kg CoCl_2_ groups compared to control, the differences did not reach statistical significance. Overall the kidneys from the group treated with 10 mg/kg ZnCl_2_ were the only group that had significantly preserved renal architecture and reduced proximal renal tubular damage (Table 1 and Fig 3A). This preservation of renal architecture may contribute to improved long term renal function.

**Table 1:** Jablonski score of rat kidneys after IRI. A grading scale of 0–4, as outlined by Jablonski et al.[10] was used for the histopathological assessment of damage to the proximal tubules induced by ischemia and reperfusion. Rats preconditioned with 10 mg/kg zinc had the lowest average Jablonski score after 60 minutes of renal ischemia followed by 7 days of reperfusion.

Group	Primary Jablonski Score for individual rats	Average Jablonski score
Saline control (n = 4)	3	2.75
	3	
	3	
	2	
Cobalt (30mg/kg) (n = 3)	3	2.67
	1	
	4	
ZnCl_2_ (5mg/kg) (n = 5)	2	2.60
	4	
	4	
	2	
	1	
ZnCl_2_ (10mg/kg) (n = 4)	1	2.00
	4	
	2	
	1	
ZnCl_2_ (30mg/kg) (n = 4)	1	3.00
	4	
	4	
	3	

### Zinc preconditioning extends the critical ischemia time

**Fig 4:**
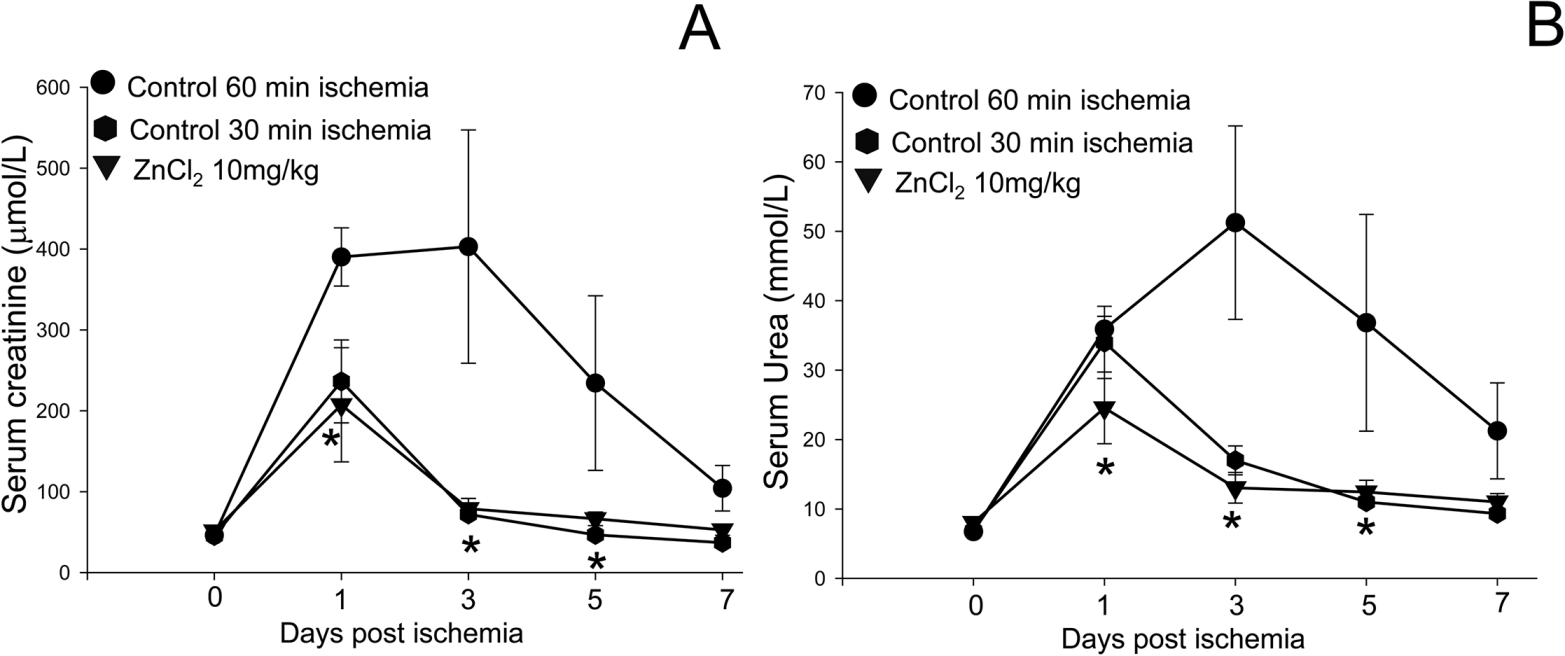
Zinc preconditioning extends the critical ischemia time. The effects of 60 minutes of ischemia with and without ZnCl_2_ preconditioning were compared to 30 minutes of ischemia in a rat model of IRI. Renal function was assessed by measurement of serum creatinine (A) and serum urea (B) in rats after 60 minutes ischemia treated with saline (●) or 10mg/kg ZnCl_2_ preconditioning (▼), or after 30 minute ischemia treated with saline (⬢). ZnCl_2_ at 10 mg/kg reduced the AKI after 60 minutes of ischemia to a level comparable to 30 minutes ischemia, thereby effectively doubling the critical ischemia time. Data are expressed as mean ± SEM for each treatment group.

Having established that ZnCl_2_ at 10 mg/kg provided the maximal protection against IRI in our model, the magnitude of the effect of ZnCl_2_ preconditioning was compared to the “safe” warm ischemia time of 30 minutes. The rise in serum creatinine and urea values induced by 60 minutes ischemia after 10 mg/kg ZnCl_2_ preconditioning was almost identical to the rise after 30 minutes of ischemia, and significantly less than in the group subjected to 60 minutes ischemia alone (Fig 4A and 4B). The increase seen in serum creatinine and urea induced by 30 minutes of ischemia is consistent with previous studies [14]. Therefore, we conclude that ZnCl_2_ preconditioning can extend the critical ischemia time from 30 to 60 minutes.

### Comparison of stimulation of HIFs by cobalt chloride and zinc chloride

**Fig 5:**
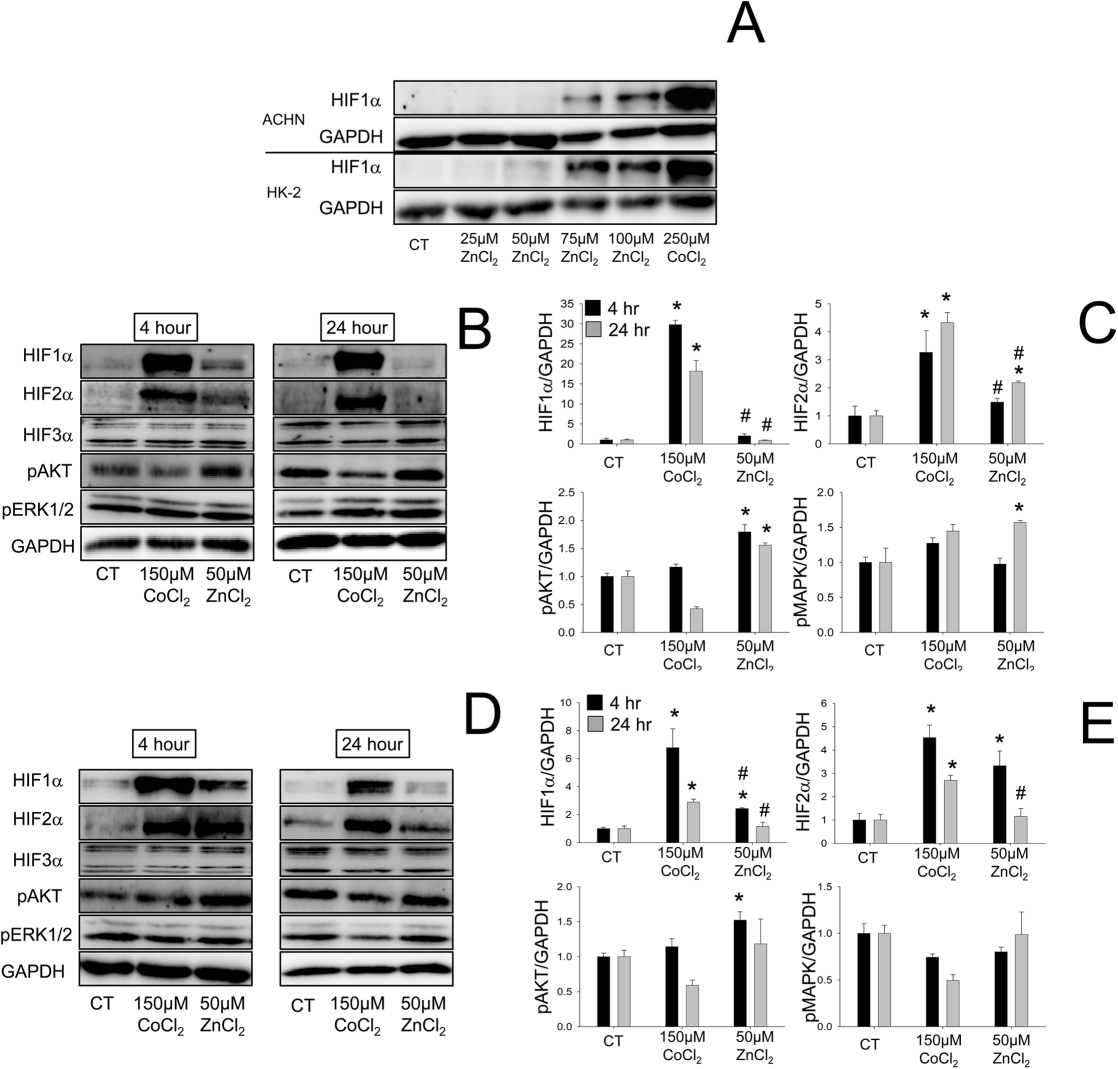
Cobalt and zinc induce HIF1α and HIF2α but not HIF3α. (A) Western blots revealed that ZnCl_2_ induces HIF1α protein in the human renal cancer cell line ACHN and the immortalized human renal tubular cell line HK-2 in a dose-dependent manner. The expression of HIF1α, HIF2α, HIF3α, phosphorylated-AKT and phosphorylated ERK1/2 proteins in ACHN (B, C) and HK-2 (D, E) cells after treatment with 150μM CoCl_2_ or 50μM ZnCl_2_ for 4 or 24 hours was measured by Western blot (B, D) and analysed by densitometry (C, E). Protein expression was normalized to glyceraldeyde-3-phosphate dehydrogenase (GAPDH), and expressed as the fold increase relative to untreated control cells. Data are mean ± SEM from at least three independent experiments. *, P<0.05 vs. control, ^#^, P<0.05 vs. 150μM CoCl_2_.

Preliminary investigation revealed that ZnCl_2_ increases the expression of HIF1α in the immortalized human proximal tubular cell line HK-2 and in the renal cancer cell line ACHN in a dose dependent manner (Fig 5A). Some toxicity at ZnCl_2_ concentrations higher than 75μM was evident from the reduction in expression of GAPDH. Therefore, in subsequent experiments a dose of 50μM ZnCl_2_ was used. In order to mimic the three-fold higher dose of cobalt (30 mg/kg, compared to 10 mg/kg ZnCl_2_) used in our *in vivo* study, a non-toxic 150μM dose of CoCl_2_ was chosen. 150μM CoCl_2_ stimulated HIF1α and HIF2α to a far greater degree than 5μM ZnCl_2_ in both ACHN and HK-2 cells with HIF expression decreasing by 24 hours (Fig 5B–5E). Interestingly, neither ZnCl_2_ nor CoCl_2_ stimulated expression of HIF3α in ACHN or HK-2 cells. Treatment with ZnCl_2_ for 4 hours stimulated phosphorylation of AKT in both ACHN and HK-2 cells (Fig 5B–5E) but activated phosphorylation of ERK1/2 only in ACHN cells at 24 hours (Fig 5B and 5C). CoCl_2_ had no effect on phosphorylation of either AKT or ERK1/2.

## Discussion

Even though the protective effect of IC on renal IRI has been shown in many animal studies, IC has not been translated to the clinic [15]. The limiting step has been the conflicting nature of the published results on IC [16, 17], with no protocol shown to be significantly superior to others. The use of IC in clinical practice may be limited due to the risk of damage to vascular structures, and the possibility of damage to the target organ in patients with co-morbidities such as diabetes or hypertension. Alternatively as no significant differences were observed between IC and remote ischemic preconditioning (RIPC) in a meta-analysis of the animal studies [15], it was hypothesized that RIPC has an equal potential for translation to the clinic. In contrast results from large-scale randomized controlled trials of RIPC focusing on patient-centred outcomes do not support its wider application [18]. Our observation that IC only marginally reduces serum creatinine and urea post renal IRI suggests that further research is warranted before embarking on clinical trials.

No study has previously compared the efficacy of IC and cobalt preconditioning in the kidney. This study has shown for the first time that renal protection afforded by CoCl_2_ is superior to IC or a combination of both after 40 minutes of warm ischemia. Treatment with CoCl_2_ has the added advantage of simplicity, and allows stimulation at any desired time point by subcutaneous injection. Our observations are consistent with a previous study which showed that CoCl_2_ administered subcutaneously at a dose of 30 mg/kg at day -1 and day 0 with 12-hr interval confers significant protection against renal IRI induced by 45 minutes of renal pedicle occlusion in rats [5]. In some instances there has been an additional protective effect of individual preconditioning techniques when administered in combination as opposed to insufficient protection when administered individually [19]. A rather surprising finding of our study was that the combination of CoCl_2_ and intermittent clamping was the worst of all the preconditioning groups. This difference was observed both in the increases of serum creatinine and urea and in the health scores. A possible mechanism is the reaction of superoxide (O_2_^-^), produced by IC, with Co^2+^ ions to produce hydrogen peroxide (H_2_O_2_), which is a toxic reactive oxygen species (ROS) [20]. Our finding emphasises the importance of pre-clinical testing of combination therapies in animal models before commencement of any human trials.

Despite the beneficial effect of CoCl_2_, the “Quebec beer-drinkers cardiomyopathy” observed in the 1960s and the associated toxicity [6] have rendered cobalt unsuitable for human use. While studies are limited, the effect on renal IRI of a related metal ion Zn^2+^, which is routinely used as a dietary supplement, are conflicting. Two decades ago, Hegenauer and co-workers showed that intravenous treatment with Zn^2+^ (2.5 mg elemental Zn/kg, equivalent to 5 mg ZnCl_2_/kg) 30 minutes prior to warm ischemia produced a significant improvement in renal function in rabbit kidneys [21]. In contrast, a decade ago Ogawa and co-workers concluded that a single intraperitoneal injection of 20 mg/kg zinc sulphate had only a minor protective effect against renal IRI induced by 30 minutes of ischemia in rats [22]. The current study has demonstrated that the efficacy of ZnCl_2_ pre-treatment against renal IRI is dose dependent. The lowest dose of 5 mg/kg ZnCl_2_ did not offer any protection and, although the rats treated with the highest dose of 30 mg/kg ZnCl_2_ displayed no signs of toxicity or discomfort, there was a reduction in renal protection post IRI compared to the optimal dose of 10 mg/kg. Therefore we concluded that at low doses ZnCl_2_ may not reach the therapeutic threshold and at higher doses, ZnCl_2_ may become ineffective due to toxicity.

Animal health scoring is typically used to assess animal well-being post-surgery. In this study the sickness score peaked early and then recovered quickly in all preconditioned rats but did not correspond with the serum creatinine and urea values. Alternatively weight loss, measured as a percentage decline from initial weight, is another commonly used criterion of animal health, as rats with acute renal failure will experience reduced appetite. While not statistically significant, a trend was observed for rats preconditioned with 10 and 30 mg/kg ZnCl_2_ to have reduced weight loss compared to control rats at all time points, and an earlier return to weight gain compared to the other groups.

The observation that IRI associated with 60 minutes of ischemia after preconditioning with 10 mg/kg ZnCl_2_ was equivalent to the “safe” 30 minutes of warm ischemia [2] suggests that ZnCl_2_ preconditioning can effectively double the safe warm ischemia time from 30 minutes to 60 minutes. If translated to the clinic this discovery will have significant implications for the management of kidney cancer and increase the proportion of cancers that can be treated with nephron-sparing PN rather than radical nephrectomy [1].

CoCl_2_ strongly induces expression of the hypoxia-inducible transcription factors (HIFs) which are involved in a cell’s ability to adapt to low oxygen (hypoxia) [5] and this induction may be one mechanism by which CoCl_2_ protects against IRI [5, 23]. More recently, several pharmaceutical companies have developed potent HIF-inducing prolyl-hydroxylase (PHD) inhibitors which have been shown to protect against renal IRI [24]. The effects of Zn^2+^ ions on the induction of HIF1α are contradictory and vary between tissues. The only study using renal cancer cells (RCC4) demonstrated that Zn^2+^ down-regulates expression of HIF1α and its downstream target VEGF *in vivo* and *in vitro* [25]. The effects of Zn^2+^ ions on HIF2α and HIF3α induction had not been investigated previously. The present study has shown for the first time that Zn^2+^ induces expression of HIF1α and HIF2α but not HIF3α in the renal cancer cell line ACHN and the immortalized normal tubular cell line HK-2.

Several studies have highlighted the complexity of IRI and indicated that HIFs are not the only important pathway. Thus, although the powerful PHD inhibitor hydralazine stimulates HIF1α expression, it does not protect against renal IRI [26]. Moreover, deletion of the HIF1α gene from cortical neurons does not diminish the protection from hypoxic ischemic injury, nor does it prevent the protective effect of PHD inhibition [27]. Also, while Co^2+^ and hypoxia both induce HIF1α and HIF2α, the overlap in the induction of gene expression is very limited [28]. Furthermore, Co^2+^ and Zn^2+^ ions induce expression of genes independently of either HIF1α or HIF2α induction [29, 30], and Zn^2+^ can activate transcription factors other than HIFs [29, 31]. Further studies are warranted to clarify the role of HIF1α and HIF2α activation in Zn^2+^-mediated protection in renal IRI. There is also a possibility of involvement of other proteins in zinc-induced protection against renal IRI. One such group of candidate proteins are metallothioneins which are cysteine-rich polypeptides of 6–7 kDa in size. A connection between their antioxidant activity and protective effects against oxidative stress is well established [32]. Interestingly a recent study found that although zinc supplementation (oral 60 mg per day) did not increase serum metallothioneins in humans there was a significant positive correlation between metallothioneins and kidney function (as measured by glomerular filtration rate), which was influenced by zinc supplementation [33].

Zn^2+^ ions have been shown to activate signalling pathways including MAPK and PI3K that are involved in cellular proliferation and survival in various cell types [29, 34, 35]. The current study demonstrates the ability of Zn^2+^ to activate phosphorylation of AKT in HK-2 and ACHN cells and the phosphorylation of ERK1/2 in ACHN cells. In contrast although Co^2+^ was able to induce expression of HIF proteins, it did not stimulate phosphorylation of either AKT or ERK1/2. The protective effect of Zn^2+^ against IRI in rat neonatal cardiomyocytes was previously shown to involve activation of the PI3K/Akt signaling pathway [36]. The *in vitro* data presented here support a role for Zn^2+^ in the activation of these pathways to protect against renal IRI.

In conclusion, Zn^2+^ preconditioning is able to protect against renal IRI in a rat model of IRI. PN is currently under-utilised because of concerns about the warm ischemia time. Although a number of drugs and agents have been shown to improve renal function and provide cytoprotection *in vitro* and *in vivo* against ischemic models of acute renal failure, all have failed in human trials and none have progressed to the clinic. However, a doubling of the currently accepted critical ischemia time through Zn^2+^ preconditioning may cause a transformational change in the management of kidney cancers.

## Supporting information

S1 File (XLSX)

## Abbreviations

IRI: ischemia reperfusion injury
HIF: hypoxia inducible factor
IC: intermittent clamping
PHD: prolyl hydroxylase
ROS: reactive oxidative species
RIPC: remote ischemic preconditioning
